# 

**Published:** 1889-09

**Authors:** 


VOL. XIX. SEPTEMBER, 1889. NO. 9

### Southern Medical Record.

EDITORS:

T. S. POWELL, M. D. R. C. WORD, M. D.

COLLABORATORS:

J. McF. Gaston, M. D.,
Julian J. Chisolm, M. D.,
W. P. Nicolson, M. D.,
E. Van Goidtsnoven, M. D.,
P. R. Cortelyou, M. D.,
Lindsay Johnson, M. D.,
G. G. Roy, M. D.,
A. G. Hobbs, M. D.,
W. S. Elkin, M. D.,
W. A Crow M. D.,
Jno Thad Johnson, M. D.,
K. P. Moore, M. D.

Address R. C. Word, M. D. Managing Editor, Atlanta, Ga.

Published and entered as Second Class Matter at Atlanta, Ga.

